# Breast Cancer Diagnosis Method Based on Cross-Mammogram Four-View Interactive Learning

**DOI:** 10.3390/tomography10060065

**Published:** 2024-06-01

**Authors:** Xuesong Wen, Jianjun Li, Liyuan Yang

**Affiliations:** School of Mechanical and Electrical Engineering, China Jiliang University, Hangzhou 310018, China; s21010811030@cjlu.edu.cn (X.W.); p21010854118@cjlu.edu.cn (L.Y.)

**Keywords:** bilateral mammograms, deep learning, breast cancer, computer-aided diagnosis, interpretable classifier

## Abstract

Computer-aided diagnosis systems play a crucial role in the diagnosis and early detection of breast cancer. However, most current methods focus primarily on the dual-view analysis of a single breast, thereby neglecting the potentially valuable information between bilateral mammograms. In this paper, we propose a Four-View Correlation and Contrastive Joint Learning Network (FV-Net) for the classification of bilateral mammogram images. Specifically, FV-Net focuses on extracting and matching features across the four views of bilateral mammograms while maximizing both their similarities and dissimilarities. Through the Cross-Mammogram Dual-Pathway Attention Module, feature matching between bilateral mammogram views is achieved, capturing the consistency and complementary features across mammograms and effectively reducing feature misalignment. In the reconstituted feature maps derived from bilateral mammograms, the Bilateral-Mammogram Contrastive Joint Learning module performs associative contrastive learning on positive and negative sample pairs within each local region. This aims to maximize the correlation between similar local features and enhance the differentiation between dissimilar features across the bilateral mammogram representations. Our experimental results on a test set comprising 20% of the combined Mini-DDSM and Vindr-mamo datasets, as well as on the INbreast dataset, show that our model exhibits superior performance in breast cancer classification compared to competing methods.

## 1. Introduction

According to 2023 statistics, breast cancer is the most prevalent cancer type among women in the United States, with an estimated 297,790 new cases, accounting for 31% of all new cancer cases, and an expected death toll of 43,170 [1]. These figures highlight the significant threat that breast cancer poses to women’s health, underscoring the importance of its prevention and early screening. Mammography screening, essential for breast cancer prevention, typically comprises two ipsilateral views of each breast: the bilateral cranio-caudal (CC) view, a top-down perspective, and the mediolateral oblique (MLO) view, angled from the chest center toward the armpit at 45 to 50 degrees [2,3]. In addition to analyzing and comparing these two views, radiologists also compare ipsilateral views (the CC view and MLO view of the same breast) and bilateral views (the same view of both breasts), as such comparisons provide a richer set of feature information [4], facilitating the search for global structural distortions, local tumors, and calcifications, thereby enabling a comprehensive analysis of each breast [5]. For each patient, four high-resolution mammographic images of the left and right breasts are taken. Any detected abnormalities will require further diagnostic imaging and tissue biopsy for confirmation. Based on the standards of the American College of Radiology’s Breast Imaging Reporting and Data System (BI-RADS), radiologists perform a standardized assessment for each screening mammogram, providing specific follow-up recommendations based on the BI-RADS score for each patient [6].

In contemporary practice, deep learning methods have rapidly evolved in the field of medical image analysis and have achieved significant results [7]. Many medical fields now employ Computer-Aided Detection (CAD) systems, providing radiologists with rapid, objective recommendations that enhance clinical decision-making during large-scale breast cancer screenings. The prevailing approach involves using multi-view image information as the input for models. For works that use ipsilateral views as model inputs, the CC and MLO views are used for feature extraction for cancer diagnosis [8,9], but each view extracts features independently and then combines them, resulting in a lack of information interaction between views, leading to the loss of inter-view information relationships. In 2021, van Tulder et al. [10] introduced an inter-view attention mechanism for information transfer between views. This method allows the model to connect different views at a more detailed spatial feature level, rather than merely concatenating at a global feature level. In 2022, Chen et al. [5] proposed the Local Co-occurrence and Global Consistency method, which not only completed the information interaction between views but also introduced inter-view similarity learning. In 2023, the CVR-RCNN network framework [11] was proposed. It aims to transfer visual and geometric information between two views, thereby enhancing tumor detection in one view. In 2023, Wang et al. [12] introduced DCHA-Net, employing Hybrid Attention in their Hybrid Attention modules for feature interaction and registration between views, thereby mitigating the feature misalignment problem between dual views. Nguyen et al. [13] introduced DIVF-Net, a network that first fuses features from bilateral views of the same breast. This process is followed by the element-wise addition of the resultant fused features to the feature map of the primary view, thereby enhancing the information of the primary view. In 2021, Yan et al. [14] proposed a unified Siamese network for simultaneous patch-level mass or non-mass classification and dual-view mass matching. In 2018, Wang et al. [15] introduced the MV-DNN approach, in which LSTM is utilized to effectively aggregate data from dual views of a unilateral breast, integrating contextual information from both views. In 2016, Bekker et al. [16] proposed the MV-NN method, which employs view-level classifiers for individual view classification judgments, followed by a neural network layer to integrate decisions from the two view levels. Although these methods demonstrated good performance, they only utilized information from dual views, neglecting the feature information of the patient’s contralateral breast and the interactive information between breasts.

Regarding the work using bilateral four-view mammograms as model inputs, in 2021, Li et al. [17] developed a multi-stream network architecture to integrate four views from bilateral breasts, enabling the network to independently learn features from each view. In the network’s later stages, features from different views are concatenated, thereby enhancing classification performance. While this approach demonstrates good performance, it does not fully exploit the latent relationships between bilateral breasts. Nguyen et al. [18] proposed a two-stage multi-view model for breast imaging. In the first stage, features are extracted separately from the left and right breasts. In the second stage, these features are averaged and input into separate BI-RADS and density classifiers using LightGBM. The final classification output is determined by taking the maximum value of the predictions from both breasts. Chen et al. [19] introduced the MVT model, which utilizes four views of bilateral mammograms as inputs. This model segments the four views into multiple patches, extracts features independently from each patch, and employs Global Transformer blocks to analyze the relationships between patches. However, due to the non-rigid nature of breasts, morphological variations across different views present significant challenges, making it difficult for the model to capture features directly related to cancer. In 2022, Lopez et al. [4] proposed the PHYSEnet model. They employed a parameterized hypercomplex neural network capable of handling breast cancer classification by capturing the intrinsic correlations between different views and using a shared encoder to analyze all four views (left and right CC and MLO), thus achieving inter-breast feature interaction. However, simply inputting all four views into a shared backbone without feature registration and contrastive work can lead to feature misalignment learning in the model.

Inspired by the limitations of these previous works, we have pioneered an approach to perform correlation and contrastive learning between the features of bilateral mammogram views. Our method requires only image-level labels to achieve accurate and robust classification of the entire mammograms of both the left and right breasts. Additionally, our approach utilizes the four-view feature information from bilateral mammograms. We have also successfully addressed the issue of incorrect feature matching between the left and right mammograms. Furthermore, our approach integrates joint and contrastive learning of cancerous and normal tissue features. This method enhances the similarity between corresponding features in the left and right mammograms while amplifying the differences between dissimilar features. Consequently, the model more effectively distinguishes between normal tissue and malignant breast lesions. Moreover, our model facilitates local feature interaction learning with the global region of the contralateral feature map, improving the classification of benign and malignant breasts. This four-view diagnostic method aligns with the practice of physicians who both analyze ipsilateral views and compare contralateral views during the diagnostic process. Our model demonstrates excellent classification performance, greatly enhancing the model’s robustness and generalizability, and achieves competitive results. In summary, our contributions are as follows:

1. We propose a novel approach to the associative matching learning of local features across global regions of bilateral mammograms, achieving feature interaction consistency and complementarity between them, effectively alleviating the issue of misaligned feature matching, and significantly enriching the information content of the related features.
2. We devised a novel joint contrastive learning strategy for bilateral mammograms capable of capturing the correlational characteristics of each local region between bilateral breast feature maps to maximize similarity. Concurrently, it facilitates the contrastive learning of distinct features to maximize feature disparity. This approach aids the model in identifying the features of cancerous regions and distinguishing them from those of normal tissue, and furthermore, it precludes the occurrence of misaligned feature learning.
3. We evaluated the performance of our proposed FV-Net model on a 20% test set of a combined dataset comprising Mini-DDSM [20] and Vindr-mamo [21], as well as on the INbreast dataset [22]. The experimental results confirm that our method outperforms competing methods on multiple datasets.

## 2. Related Work

Deep learning models are now widely used in the diagnosis of breast cancer, with two primary approaches to classification. The first approach requires a binary segmentation mask during training. Ru et al. [23] proposed the Att-U-Node for automatically segmenting breast tumors. This network utilizes an attention module to guide a framework based on neural ODEs, addressing several challenges faced by popular deep neural networks, such as large parameter counts, a lack of interpretability, and issues with overfitting. Tsochatzidis et al. [24] introduced a method that integrates tumor segmentation information from mammograms into a Convolutional Neural Network (CNN), aiming to improve the diagnosis of breast cancer in mammograms. Furthermore, Ghosh et al. [25] designed an innovative automatic mammogram segmentation method. This method employs Intuitionistic Fuzzy Soft Sets (IFSS) and multi-granularity rough sets. It introduces a novel hybrid soft computing approach, which is integrated into the segmentation process. This enhances the early detection of breast cancer. Sarangi et al. [26] developed a model using a single-layer Legendre neural network, which was trained with a Block-Based Normalized Sign-Sign Least Mean Square (BBNSSLMS) algorithm to enhance performance. However, the aforementioned methods require experienced radiologists to annotate regions of interest (ROIs) at the pixel level, significantly increasing data annotation costs. The second approach no longer requires pixel-level ROI annotations, and many of its methods employ CNN technology to diagnose breast cancer [27,28,29,30,31]. For instance, Albashish et al. [30] employed a transfer learning model based on the Visual Geometry Group’s 16-layer deep model architecture (VGG16), outperforming the latest classical machine learning algorithms in breast cancer diagnosis. Heenaye-Mamode Khan et al. [32] used a pretrained ResNet50 model to develop an enhanced deep learning model, achieving an accuracy of 88%. Although these methods have achieved significant success in medical image processing and play an important role in breast cancer screening, due to the limited receptive field of convolutional kernels, CNN methods struggle to capture the interactive information of bilateral mammograms and thus leverage their potentially valuable information. The limitations of CNNs make it difficult to utilize global information. Our proposed method not only employs CNNs to extract features from bilateral mammograms but also realizes local-to-global area feature attention correlation learning between the two breasts, overcoming the limitation of the CNN’s local receptive field. Additionally, in terms of model design, current works [5,8,9,10,11,12,14,33,34,35,36] mainly focus on improving the diagnostic accuracy for individual breasts, with only a few methods designing models for four-view analysis [4,18,19,37]. However, these models fail to fully explore the relationship between bilateral mammograms. Therefore, it is both urgent and crucial to enhance the diagnostic capabilities of bilateral multi-view breast models for clinical decision-making. Our proposed method leverages information from four views to diagnose bilateral mammogram images simultaneously, enhancing the model’s classification capability by utilizing the latent relationship between the bilateral mammary glands. This approach culminates in an end-to-end model capable of concurrently predicting the probability of breast cancer in both breasts.

## 3. Materials

### 3.1. Data Collection

**Figure 1:**
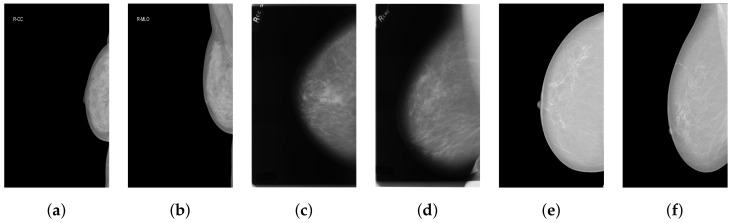
Examples from the training and test datasets of mammography images. (**a**,**b**) Vindr-mamo mammography images; (**c**,**d**) Mini-DDSM mammography images; (**e**,**f**) INbreast mammography images.

The datasets used in this study include the Mini-DDSM dataset, the Vindr-mamo dataset, and the INbreast dataset. Each breast in the datasets has CC (cranio-caudal) and MLO (mediolateral oblique) mammographic X-ray views, with each case comprising four mammograms. Due to the insufficient number of cases and their label distribution in either Mini-DDSM or Vindr-mamo alone, which did not meet our requirements for various case types, we decided to merge these two datasets. Together, the Mini-DDSM and Vindr-mamo datasets include a total of 6952 cases of full-field digital mammographic X-ray images. However, two cases from the Vindr-mamo dataset are missing some mammograms, leading to a final count of 6950 cases. Figure 1 shows a few mammography images from the datasets we used.

**Mini-DDSM:** The Mini-DDSM dataset contains 7808 mammograms. The breast classification labels in this dataset are categorized as benign, cancer, and normal. In our study, we classify normal and benign cases as negative (label: 0) and cancer cases as positive (label: 1). The dataset includes 679 cancer cases, 671 benign cases, and 602 normal cases.

**Vindr-mamo:** The original Vindr-mamo dataset contains 5000 cases, but only 4998 cases are actually usable. This dataset does not directly provide breast classification labels but rather offers BI-RADS scores ranging from 1 to 5 for each full-field digital mammographic X-ray image. In our study, we consider cases with BI-RADS scores of 1 to 3 as negative and those with scores of 4 and 5 as positive.

**INbreast:** The INbreast dataset consists of 115 cases (410 images), of which only 75 cases are composed of four views from bilateral mammograms; thus, these 75 cases were used as the test set. This dataset provides various types of annotations, including BI-RADS classification scores, masks for the segmentation of masses or calcifications, and other data annotations. We primarily determine the labels based on the BI-RADS rating scale: BI-RADS levels 1–3 are considered negative, while 4–6 are deemed positive.

### 3.2. Data Preprocessing

Given the abundance of irrelevant background information in mammograms and the fact that the lesion area occupies only a small part of the entire mammographic image, combined with the certain obscurity of the breast lesion area, it is necessary to preprocess the mammography images to enhance the contrast and recognizability of different features within the breast area. We employ various processing techniques, including Breast Region Detection (BRD), Contrast-Limited Adaptive Histogram Equalization (CLAHE), and truncated normalization.

#### 3.2.1. Breast Region Detection (BRD)

**Figure 2:**
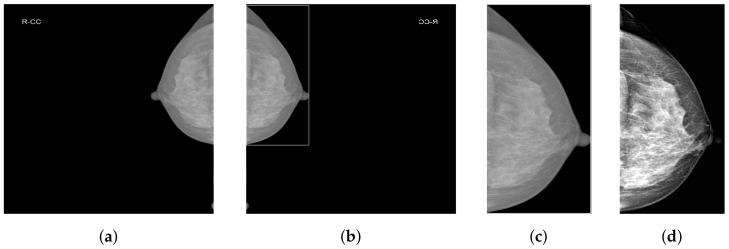
The data preprocessing procedure. (**a**) The original image; (**b**) the image processed with BRD; (**c**) the image processed with BRD and cropping; (**d**) the image processed with BRD, cropping, CLAHE, and truncated normalization.

As shown in Figure 2a, mammograms contain a large amount of irrelevant black background pixel information. Removing background pixels unrelated to classification allows the model to focus only on the features of the breast area. We employ our custom-designed Breast Region Detection (BRD) method to preprocess the mammograms. First, the module conducts orientation detection on all mammographic images to ensure all images are oriented toward the right-hand side, thus preventing the model from being influenced by varying image orientations during the learning process. It uses a GaussianBlur with a 5 × 5 kernel for image smoothing to reduce noise. It then utilizes OTSU thresholding to automatically determine the optimal threshold for the mammographic images and performs binarization, significantly separating the breast area from the background. The findContours function is used to detect the edges of the breast area in the binary image, eventually obtaining the rectangular area of the largest contour of the breast area, as shown in Figure 2b.

#### 3.2.2. Contrast-Limited Adaptive Histogram Equalization and Truncated Normalization

Due to the extremely subtle differences between healthy and pathological tissues in the breast, we enhance local pixel contrast by applying Contrast-Limited Adaptive Histogram Equalization (CLAHE) [38]. After analysis and comparison, we set the clipLimit parameter value to 1.0. Additionally, considering the presence of extremely dark and bright areas within the breast, we empirically set two percentile thresholds at 5% and 99%. This means ignoring the lowest 5% and the highest 1% of all pixel values in the image. The clipped pixel values are then normalized, which reduces image variations caused by noise or non-representative features and further enhances image contrast. Figure 2d presents the final effect of data preprocessing.

### 3.3. Data Upsampling

Due to the significant imbalance in the dataset distribution, it is crucial to prevent the model from disproportionately focusing on the majority negative sample categories, thereby neglecting the minority positive categories. Therefore, it is necessary to upsample cases with cancer in both breasts as well as cases with cancer in only one breast. When integrating the Mini-DDSM and Vindr-mamo datasets, we do not simply merge them into a single dataset. Instead, we rearrange the data by individual cases to ensure a uniform distribution of different case categories, thereby better addressing the issue of data imbalance. Subsequently, we allocate 20% of the processed dataset to the test set and the remaining 80% to the training set, with the specific data distribution detailed in Table 1.

As shown in Table 2, after upsampling the data in the training set, we obtain data for 4629 patients with no cancer in either breast, 2749 patients with cancer in both breasts, and 1880 patients with cancer in only one breast. This approach ensures that the sum of the number of cases with cancer in both breasts and the number of cases with cancer in only one breast is equal to the number of cases where both breasts are normal.

## 4. Methods

### 4.1. Problem Statement

We established a training dataset that includes bilateral four-view mammographic images and applied weakly supervised learning to the dual views of each breast per case, indicating that the entire mammogram requires only an image-level label. The labels for different views of the same breast are identical, denoted by $D={\left\{\left(R_{i}^{m},R_{i}^{c},L_{i}^{m},L_{i}^{c},y_{i}\right)\right\}}_{i=1}^{\left|D\right|}$, where $R_{i}^{m}$ and $R_{i}^{c}$ represent the MLO (mediolateral oblique) and CC (cranio-caudal) views of the patient’s right breast, respectively, and $L_{i}^{m}$ and $L_{i}^{c}$ represent the MLO and CC views of the left breast for the same patient. The index i denotes the patient number. The label $y_{i}\in Y=\left\{0,1\right\}$, where 1 indicates cancer (positive) and 0 indicates normal (negative). Here, the label $y_{i}$ refers to the annotations made by doctors indicating whether the mammogram is of a cancerous or normal breast. The test set is defined in the same manner. The ultimate goal is to accurately predict the probability of cancer in each patient’s left and right breasts.

### 4.2. Motivation

**Figure 3:**
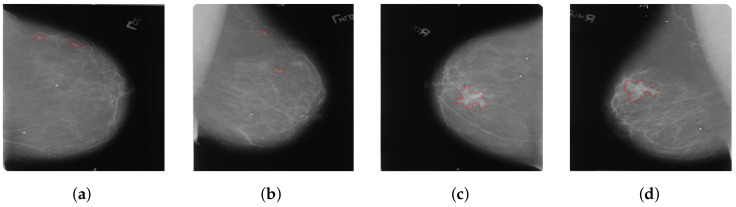
(**a**) The left CC (cranio-caudal) view; (**b**) The left MLO (mediolateral oblique) view; (**c**) The right CC view; (**d**) The right MLO view. The areas within the red bounding boxes represent the lesion regions of the mammograms.

Considering that most existing methods primarily focus on the analysis of dual-view mammography images of a single breast, only a few studies have explored feature learning across four-view mammography images of bilateral breasts. We have found that the existing four-view methods typically enable only simplistic and direct interactions of features between bilateral mammography images. These interactions are clearly insufficient for complex four-view analysis. Our approach addresses a critical issue in the analysis of bilateral breast four-view mammography: when extracting features using four-view mammography images of bilateral breasts, the uncertainty in the size and location of lesions in the bilateral breasts often leads to incorrect feature matching, as shown in Figure 3. This issue can make it difficult for the model to effectively distinguish between cancerous and non-cancerous tissues, thereby severely affecting the model’s learning performance. We introduce a novel local-feature-matching learning mechanism for bilateral breasts. Additionally, we developed a contrastive joint learning method for bilateral breasts. These innovations effectively alleviate the issue of incorrect feature matching in bilateral breasts and significantly enhance the model’s ability to differentiate between features.

One potential solution is to employ semantic segmentation methods [39,40] to explicitly delineate the lesion areas’ location and size in the breast for the model. However, this approach necessitates fine-grained pixel-level annotations of mammography images by doctors, implying substantial manual annotation costs and the risk of errors due to repetitive, intensive labor. Observing the characteristics of the dataset, we noted that the bilateral breast distribution in cases can be categorized into three scenarios: (1) both breasts are normal; (2) both breasts are cancerous; (3) only one breast is cancerous. Therefore, to address cross-breast feature learning in these distinct scenarios, we introduced a bilateral breast local-feature-matching learning mechanism and achieved inter-breast similarity feature correlation learning, as well as dissimilar feature contrastive learning, thus resolving the aforementioned issues. Our proposed Four-view Correlation and Contrastive Joint Learning Model (FV-Net) primarily comprises the following modules:

1. Pretrained truncated EfficientNet-b0: used for extracting features from both breasts.
2. Cross-Mammogram Dual-Pathway Attention Module: used for calculating local-feature-matching relationships between the left and right breast feature maps.
3. Bilateral-Mammogram Contrastive Joint Learning: used for the joint contrastive learning of the interaction feature maps.
4. Classifier and loss function: includes fully connected layers for the left and right breasts, BCE loss, and global average pooling.

### 4.3. Pretrained Truncated EfficientNet-b0 Module

**Figure 4:**
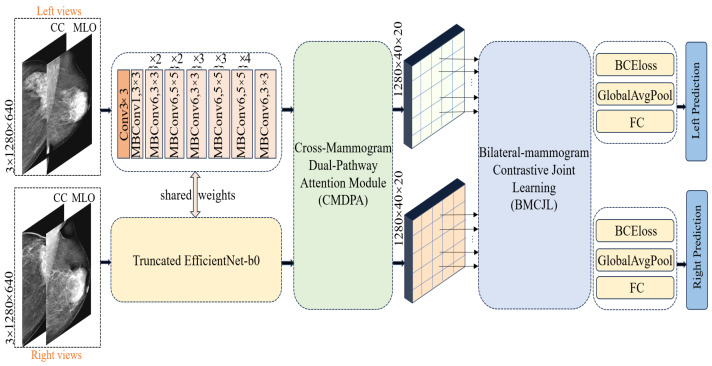
Our proposed FV-Net framework utilizes a shared modified truncated EfficientNet-b0 for feature extraction and employs the CMDPA to reshape the fea ture maps, along with the BMCJL module for four-view local feature joint contrastive learning.

In processing unilateral breast images, we first concatenate the CC (cranio-caudal)-view and MLO (mediolateral oblique)-view mammographic images of the left breast for each case, applying the same procedure for the right breast. The concatenated mammographic images of the left and right breasts are then fed into a shared feature extractor. We opt for a truncated version of EfficientNet-b0, pretrained on ImageNet-1K [41], as our feature extractor; detailed information about its architecture can be found in the “shared weights” section in Figure 4. Diverging from the classic EfficientNet-b0, we removed the final fully connected layer and pooling layer, retaining only the feature extraction module to preserve more spatial information. The feature extractor reduces the size of the original image by 1024 times, outputting feature maps of the left and right breasts with dimensions of 40 × 20 and a feature channel count of 1280. Each pixel point corresponds to a receptive field of size 32 × 32 in the original image; this is specifically achieved through the following equations:

(1) $$F_{L}=f\left(L_{i}^{m},L_{i}^{c}\right)$$

(2) $$F_{R}=f\left(R_{i}^{m},R_{i}^{c}\right)$$

### 4.4. Cross-Mammogram Dual-Pathway Attention Module

Our method incorporates an attention block [42] comprising three core elements: *Q* (Query vector), *K* (Key vector), and *V* (Value vector). The Query vector represents the feature item currently being processed, the Key vector is used for matching with the Query vector, and the Value vector contains the actual content of information. This content, after being weighted by attention, is used to construct the final output. *Q*, *K*, and *V* are typically represented as feature tensors of the shape $R^{C\times D}$. The computation of relations is achieved by computing the dot product of the Query with all Key items, dividing by $\sqrt{C}$ (where *C* represents the dimension of the Key vector), and then applying the softmax function to obtain attention weights for the input elements. These weights are multiplied by the Value vector *V* to obtain a weighted summary containing information weighted based on the association between the Query and the Keys. The computation equation is as follows:

(3) $$F\left(Q,K,V\right)=\mathrm{softmax}\left(\frac{QK^{T}}{\sqrt{C}}\right)V$$

**Figure 5:**
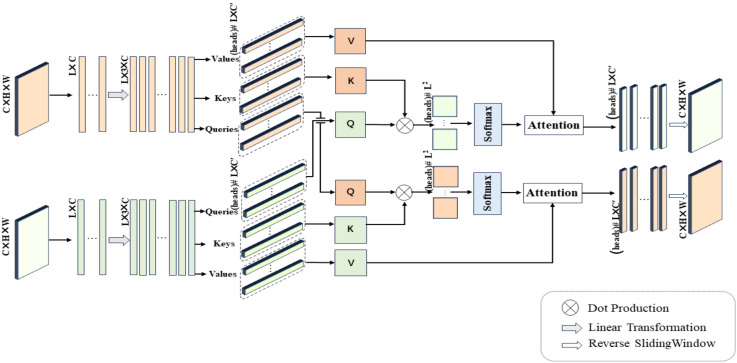
Detailed architecture of Cross-Mammogram Dual-Pathway Attention Module, (heads) # L × C’ represents numerical units with the total number of heads and a size of L × C’, and the others are similarly defined.

Next, we describe an innovative application of Equation (3) in our study, namely, the cross-mammogram local multi-head interaction relationship, as detailed in Figure 5. As illustrated in Figure 5, our proposed Cross-Mammogram Dual-Pathway Attention Module adopts a dual-path architecture, aiming to capture the feature correlation and matching information between bilateral mammograms. Taking the original high-dimensional feature maps of bilateral breast images, $F_{L},F_{R}\in R^{B\times C\times H\times W}$, as input, we first reshape the input features into the shape of (B,L,C), forming the feature matrices of the left and right breasts, $A_{L}=\left[a_{1}^{L},a_{2}^{L},\ldots ,a_{n}^{L}\right]$, $A_{R}=\left[b_{1}^{R},b_{2}^{R},\ldots ,b_{n}^{R}\right]$. Subsequently, through two linear transformation branches, $A_{L}$ and $A_{R}$ are mapped into $P_{L},P_{R}\in R^{B\times L\times 3C}$. By performing sliding segmentation on the channel dimension of the mapped $P_{L}$ and $P_{R}$, the Query map, Key map, and Value map $\in (B,M,L,C^{\prime })$ are generated. From these, we separate out *M* sets of $Q_{i},K_{i},V_{i}$, and $Q_{i},K_{i},V_{i}\in R^{B\times L\times d},i=0,\ldots ,M-1$, where the parameter *M* controls the number of feature attention heads in multi-head attention. Here, the parameter *d* represents the dimension of the features within each attention head. The value of *d* can be calculated by the following equation:

(4) $$d=C^{\prime }=\frac{3C}{M}$$

For each *Q* in the Query map, we compute its dot-product attention with all *Ks* in the Key map of the contralateral mammograms and employ the softmax function to obtain normalized bilateral breast local feature correlation attention weights. These attention weights are then used to weight the corresponding *Vs* in the Value map. This process is symmetrically applied to both breasts. Therefore, the relation maps for each attention head of the left and right mammography views are, respectively, calculated by the following equations:

(5) $$F_{L_{i}}^{\mathrm{mha}}\left(Q,K,V\right)=\mathrm{softmax}\left(\frac{Q_{L_{i}}K_{R}^{T}}{\sqrt{C}}\right)V_{R}$$

(6) $$F_{R_{i}}^{\mathrm{mha}}\left(Q,K,V\right)=\mathrm{softmax}\left(\frac{Q_{R_{i}}K_{L}^{T}}{\sqrt{C}}\right)V_{L}$$

In Equations (5) and (6), $Q_{L_{i}}$ and $Q_{R_{i}}$ represent the Query of each local feature attention head for the left and right mammography feature maps, respectively. $K_{L}$, $K_{R}$, $V_{L}$, and $V_{R}$ correspond to all Keys and Values within the Key maps and Value maps of the mammographic feature representations. Finally, after linear projection and feature reshaping, the weighted feature matrix obtained from each attention head is recombined into a cohesive feature map. The resulting new feature maps, denoted by $F_{L}^{\prime \prime }$, $F_{R}^{\prime \prime }\in R^{C\times H\times W}$, maintain their complete spatial structure. This facilitates the associative matching of local feature information from each mammogram with the global feature regions of the contralateral breast.

### 4.5. Bilateral-Mammogram Contrastive Joint Learning

Inspired by the diverse distribution of label combinations in bilateral breast images, in FV-Net, we innovatively design a contrastive joint loss function, introducing a novel method for local feature interaction learning between bilateral mammographic feature maps. This method aims to maximize the similarity between the most similar local features and enhance the disparity between the most dissimilar local features. As shown on the right side of Figure 4, the recombined feature maps of the left and right mammograms, $F_{L}^{\prime \prime }$, $F_{R}^{\prime \prime }$, processed by the “Cross-Mammogram Dual-Pathway Attention Module”, are divided into equally sized, non-overlapping patch blocks. Each block consists of $\frac{H\times W}{k^{2}}$ patches, where $\left\{F_{L_{i}}^{\prime \prime },F_{R_{i}}^{\prime \prime }\right\}\in R^{C\times k^{2}}$ for $i=0,\ldots ,\frac{H\times W}{k^{2}}-1$. Each patch block size is (*C*, *K*, *K*), and the feature map is divided from (*C*, *H*, *W*) into (*N*, *C*, *K*, *K*), where *N* is the number of patches, and *K* is set to 5.

We calculate the similarity between the feature maps $F_{L_{i}}^{\prime \prime }$ of each left mammographic image and the feature maps $F_{R_{i}}^{\prime \prime }$ of the contralateral mammographic image. This process is accomplished by comparing similarity scores between different patch blocks to identify pairs of patches in $F_{L}^{\prime \prime }$ and all patch blocks in $F_{R}^{\prime \prime }$ with the highest and lowest similarity scores. The patch pairs with the highest similarity scores are considered positive samples, while those with the lowest scores are considered negative samples. Specifically, when the labels of the left and right breasts indicate unilateral or bilateral cancer, we need to determine the indices of the feature patch pairs with the highest and lowest similarity, that is, the positive and negative sample pairs. However, when both breasts are normal, we only need to identify the most similar patch index pairs, that is, the positive sample pairs.

More specifically, we first normalize the given feature vectors $X_{i}$ and $Y_{j}$ (representing two patch blocks from bilateral mammograms) separately. This step is achieved by dividing each feature vector by its L2 norm (adding a small constant $\epsilon =1\times 10^{-6}$). The normalized feature vectors $X_{i}$ and $Y_{j}$ ensure consistency in length, making the subsequently calculated similarity scores reflect only directional differences. The similarity score, Sim, is obtained by calculating the dot product of the normalized feature vectors as follows:

(7) $$\mathrm{Sim}\left(X_{i},Y_{j}\right)=\frac{X_{i}}{∥X_{i}∥+\epsilon }\cdot \frac{Y_{j}}{∥Y_{j}∥+\epsilon }$$

The method for identifying positive and negative sample pairs is as follows: after calculating the similarity between all pairs of patches, we use the following Equation (8) to determine the indices of the most similar (positive sample pairs) and the least similar (negative sample pairs) patch pairs:

(8) $$j\left(i\right)=\arg\;\max\;\mathrm{Sim}\left(X_{i},Y_{j}\right)$$

(9) $$j^{\prime }\left(i\right)=\arg\;\min\;\mathrm{Sim}\left(X_{i},Y_{j}^{\prime }\right)$$

Equation (8) implements the assignment of each index *i* to index *j* of the patch $Y_{j}$ in the contralateral breast’s feature map that is most similar to the patch $X_{i}$ on this side of the breast’s feature map, thereby establishing a positive sample pair between the feature maps of the left and right breasts. Similarly, the index of the patch pair with the lowest similarity score is determined through Equation (9), thereby establishing a negative sample pair.

After determining all positive and negative sample pairs, we employ the designed joint contrastive loss function $L_{\mathrm{sim}}$ for learning. This loss function aims to reward the similarity between positive sample pairs and penalize the similarity between negative sample pairs. Its expression is as follows:

(10) $$\begin{array}{cc} L_{\mathrm{sim}}=\frac{1}{2N}\sum_{i=0}^{N-1}\left(1-I\left(y\right)\right) & \left[\exp\left(-\frac{\mathrm{Sim}(X_{i},Y_{j(i)})}{t}\right)+\exp\left(\frac{\mathrm{Sim}(X_{i},Y_{j^{\prime }\left(i\right)}^{\prime })}{t}\right)\right]\\ \; & +\frac{1}{N}\sum_{i=0}^{N-1}I\left(y\right)\exp\left(-\frac{\mathrm{Sim}(X_{i},Y_{j(i)})}{t}\right) \end{array}$$

Here, *N* represents the total number of patch blocks in the reconstructed feature map of a unilateral breast, and *t* represents the temperature parameter, which we set to 0.5. *I*(*y*) is an indicator function that is true when the labels for a pair of mammographic images are both normal. In this scenario, the loss function primarily focuses on the similarity of the most similar patch pairs. When at least one image is labeled as abnormal, the loss function takes into account the similarity of both the most similar and the least similar patch pairs. We calculate the similarity $L_{{\mathrm{sim}}_{i}}$ for each pair of patch blocks in the model and then compute their average. Our proposed joint contrastive learning loss function encourages the model to reduce differences between positive sample pairs and increase differences between negative sample pairs. This strategy facilitates the model’s ability to more easily differentiate between cancerous and non-cancerous features, thereby significantly enhancing the model’s robust classification capabilities.

### 4.6. Classifier and Loss Function

In our proposed FV-Net framework, the ultimate goal is to concurrently classify bilateral mammogram images. To this end, on top of the extracted and reconstructed feature maps, we design a dual-path classifier with non-shared weights for the classification predictions of the left and right breasts, denoted by $L_{cls}$ and $R_{cls}$, respectively. The shape of the reconstructed feature maps for the left and right breasts is (2, 1280, 40, 20). First, we perform global average pooling on each breast feature map to obtain a 1280-dimensional feature vector representation. Then, through their respective sequences of fully connected layers, followed by a sigmoid function, we predict the probability of cancer presence p for each breast, where p denotes the likelihood of malignant tumor lesions in the input images. The classification training loss functions for the left and right breasts are defined as follows:

(11) $$L_{\mathrm{cls}\_\mathrm{loss}}=-\frac{1}{S}\sum_{i=1}^{S}\left[y_{i}\log\left(P_{L_{i}}\right)+\left(1-y_{i}\right)\log\left(1-P_{L_{i}}\right)\right]$$

(12) $$R_{\mathrm{cls}\_\mathrm{loss}}=-\frac{1}{S}\sum_{i=1}^{S}\left[y_{i}\log\left(P_{R_{i}}\right)+\left(1-y_{i}\right)\log\left(1-P_{R_{i}}\right)\right]$$

During the training process, the BCE (Binary Cross-Entropy) loss is utilized to quantify the discrepancy between the predicted values $P_{L_{i}}$ and $P_{R_{i}}$ and the true value $y_{i}$. The total loss of the FV-Net model is calculated, as expressed in Equation (13), where $u_{1}$ and $u_{2}$ are the weighting coefficients, set to 5/6 and 1/6, respectively.

(13) $$L_{\mathrm{total}\_\mathrm{loss}}=u_{1}\cdot L_{\mathrm{sim}}+u_{2}\cdot \left(L_{\mathrm{cls}\_\mathrm{loss}}+R_{\mathrm{cls}\_\mathrm{loss}}\right)$$

## 5. Experiment

### 5.1. Implementation Details

First, we adjust the size of the mammography images, which consist of concatenated dual-view images of each breast for every case, to a resolution of 1280 × 640 pixels. In the dataset, 20% of the cases are allocated to a test set, while the remaining 80% are designated for the training set and are subjected to upsampling. Care is taken to ensure that data from the same patient do not appear in both the training and test sets concurrently. For the training set, we implement a series of data augmentation techniques, including the random horizontal and vertical flipping of images, applying RandomResizedCrop and adjusting the cropping area to a resolution of 1280 × 640 pixels, employing random affine transformations, conducting random color enhancement, and utilizing the RandomErasing technique. The proposed FV-Net model is implemented using the PyTorch library, with model training completed on an NVIDIA GeForce RTX 4090 GPU with 24 GB of memory. The Adam optimizer is employed, with the initial learning rate set to $1\times 10^{-4}$ and the weight decay at $5\times 10^{-4}$. A ReduceLROnPlateau learning rate scheduler is used, reducing the learning rate by a factor of 10 if there is no improvement in the loss value over 3 consecutive epochs. The loss function for the classification head is BCEWithLogitsLoss, and the batch size is set to 8. Each batch is fed with upsampled training data using a custom sampler (PatientSampler), ensuring that an equal number of cases are randomly selected from each category (cancer and normal), thereby balancing the class ratio in each batch and effectively learning the features of breast lesion areas in each iteration.

### 5.2. Evaluation Metrics

Our proposed FV-Net model is employed to predict whether a mammographic image represents a malignant or normal breast, thus presenting a classification challenge. To evaluate the performance of the model, we utilize the commonly adopted evaluation metrics for classification models in most of the literature, namely, the Area Under the Receiver Operating Characteristic Curve (AUC-ROC) and accuracy (ACC). The Area Under the Curve (AUC) of the ROC, where the ROC curve’s x-axis represents the False Positive Rate (FPR) and the y-axis represents the True Positive Rate (TPR), is a crucial metric for assessing the performance of a binary classification model. An AUC value closer to 1 indicates better model performance [43]. The calculation of accuracy (ACC) is as follows:

(14) $$\mathrm{Accuracy}=\frac{\mathrm{TP}+\mathrm{TN}}{\mathrm{FP}+\mathrm{FN}+\mathrm{TP}+\mathrm{TN}}\times 100\%$$

The True Positive Rate (TPR) and False Positive Rate (FPR) are defined, respectively, as follows:

(15) $$\mathrm{TPR}=\frac{\mathrm{TP}}{\mathrm{TP}+\mathrm{FN}}$$

(16) $$\mathrm{FPR}=\frac{\mathrm{FP}}{\mathrm{FP}+\mathrm{TN}}$$

In this context, TP (True Positive), TN (True Negative), FP (False Positive), and FN (False Negative), respectively, represent the number of malignant samples correctly identified, the number of normal samples correctly identified, the number of normal samples incorrectly identified as malignant, and the number of malignant samples incorrectly identified as normal.

### 5.3. Ablation Analysis

To evaluate the effectiveness of each component of our proposed FV-Net model, we conduct an ablation study on the well-known public mammographic X-ray image dataset INbreast. As shown in Table 3, we compare the performance of five different model variants on the INbreast dataset based on accuracy (ACC) and AUC-ROC results. These variants include (1) training on the baseline model efficientnet-b0 using raw data without preprocessing (first row); (2) training on the efficientnet-b0 baseline model with data processed by the preprocessing module (second row); (3) combining the preprocessing module with the Cross-Mammogram Dual-Pathway Attention Module (CMDPA) (third row); (4) combining the preprocessing module with Bilateral-Mammogram Contrastive Joint Learning (BMCJL) (fourth row); (5) using FV-Net, which both employs the preprocessing module and integrates the CMDPA and BMCJL (fifth row).

The results of the ablation experiments demonstrate that the baseline model without preprocessing exhibits the poorest performance, whereas the model incorporating the preprocessing module, CMDPA, and BMCJL concurrently manifests superior performance. Compared to the original baseline model, our proposed FV-Net significantly enhances the accuracy by 19.34% and the Area Under the Curve (AUC) by 0.1504. This highlights the combined efficacy of the three components: the preprocessing module, the CMDPA, and BMCJL. Specifically, the implementation of the preprocessing module alone results in an increase of 12% in accuracy and 0.0809 in AUC. Furthermore, the combined use of the preprocessing module and CMDPA, in contrast to the baseline model, leads to a 16.67% rise in accuracy and a 0.1059 boost in AUC. On the other hand, the integration of the preprocessing module and BMCJL compared to the baseline model increases the accuracy by 18.67% and the AUC by 0.103. It is noteworthy that adding the CMDPA alone leads to an increase of 4.67% in accuracy and an enhancement of 0.025 in AUC. Similarly, incorporating only the BMCJL module results in an improvement of 6.67% in accuracy and a rise of 0.0221 in AUC. When the preprocessing module, the CMDPA, and BMCJL are employed simultaneously, there is an additional improvement of 7.34% in accuracy and 0.0695 in AUC compared to the model that incorporates only the preprocessing module. The above experimental data clearly demonstrate that the data preprocessing module effectively addresses the issue of poor image quality in the original training dataset. It trims the background areas and highlights the pixels of the lesion areas, allowing the model to focus solely on the features of the breast region. Secondly, the CMDPA effectively facilitates the matching learning of local feature correlations across global regions of the breast, significantly mitigating potential misalignment issues between the features of bilateral mammogram views. Additionally, the experimental results demonstrate that the contrastive joint loss function applied by the BMCJL module effectively enables the model to learn the differences between lesion area features and normal tissue features, as well as the similarities among features within the same category. This not only facilitates the model’s ability to distinguish between lesion areas and normal tissue regions in the breast but also allows it to acquire a more comprehensive set of similar feature information.

**Figure 6:**
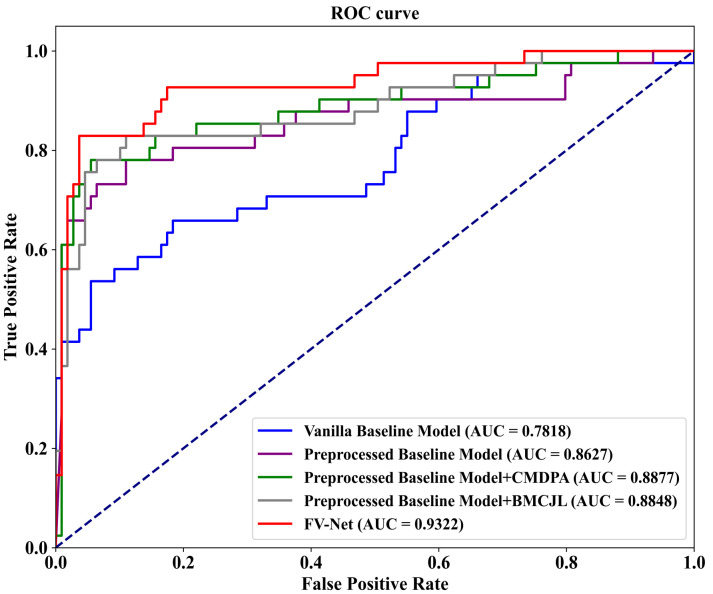
ROC curves graph from ablation study on the public INbreast dataset.

To visualize our experimental results, we plot ROC-AUC curves for five model variants on the INbreast dataset. As shown in Figure 6, these variants include (1) the Vanilla Baseline Model, corresponding to the first row on the bottom right of the graph; (2) the Preprocessed Baseline Model, corresponding to the second row; (3) the Preprocessed Baseline Model with the CMDPA, corresponding to the third row; (4) the Preprocessed Baseline Model with BMCJL, corresponding to the fourth row; (5) the complete FV-Net model, corresponding to the fifth row. These ROC-AUC curves clearly demonstrate that our experimental results are entirely consistent with the above analysis, unequivocally confirming the effectiveness of the preprocessing module, the CMDPA, and BMCJL.

**Figure 7:**
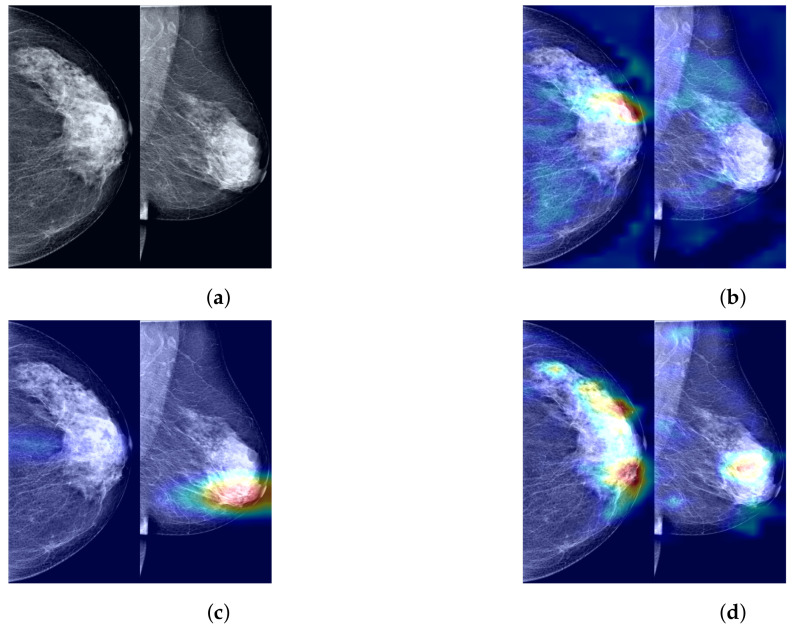
A comparison of visualization results for the most suspicious malignant tumor areas in paired CC and MLO views of the same case predicted by different method groups. (**a**) Raw image; (**b**) Preprocessed Baseline; (**c**) Preprocessed Baseline Model with BMCJL; (**d**) FV-Net.

To further demonstrate the role of the components that we propose in the process of predicting the probability of breast cancer through our model, clarify the extent to which our method enhances the model’s ability to identify tumor regions, and improve the model’s credibility by visualizing and explaining the rationale behind model predictions, we utilized Grad-CAM [44] to visualize the most suspicious cancerous areas predicted by different method groups. Grad-CAM, which is used as a visualization explanation method to generate model focus areas, highlights key areas for classification decisions by computing the gradient of the cancer probability prediction relative to the feature maps of the last convolutional layer. These gradients indicate the contribution of each unit (i.e., each pixel or region) within the feature map to the final cancer probability prediction, with the brightest areas representing the most suspicious tumor regions contributing the most to the final cancer probability prediction. As shown in Figure 7b, when using only the Preprocessed Baseline Model for predicting the probability of cancer, the absence of interactive learning of bilateral breast feature maps prevents the association and comparative learning of bilateral mammogram views, and thus, the model is easily distracted by some irrelevant features, with the model’s attention only focused on small areas at the tumor edge. As shown in Figure 7c, after solely incorporating the joint contrastive learning module, the model focuses on the local area of the tumor rather than the peripheral areas. Moreover, compared to Figure 7b, the area of attention of the model is more comprehensive, covering a larger extent of the tumor area. This is attributed to the joint contrastive learning of the features of bilateral mammogram views. As shown in Figure 7d through Grad-CAM, by integrating the joint contrastive module (BMCJL) with the Cross-Mammogram Dual-Pathway Attention Module (CMDPA), the model is capable of more comprehensively and accurately focusing on and identifying tumor regions in both CC and MLO views. This not only enables the model to effectively distinguish between cancerous regions and normal tissue areas but also mitigates the issue of incorrect feature space matching.

### 5.4. Comparison with Other Methods

**Figure 8:**
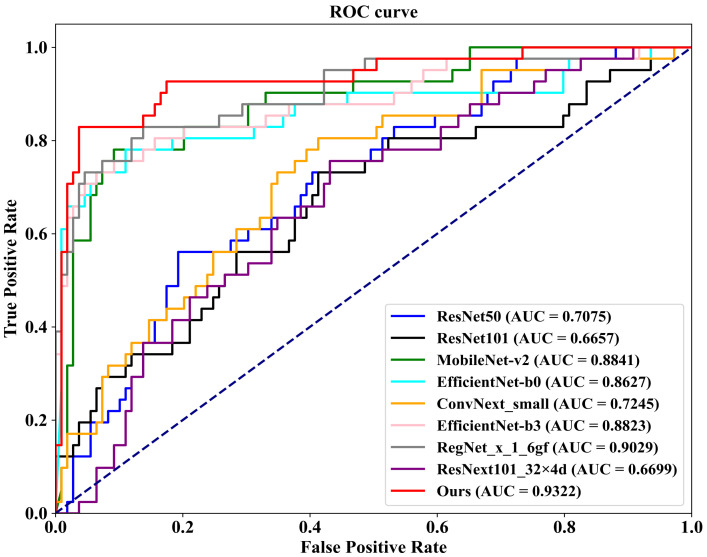
The ROC curves comparison of the classification performance of FV-Net and eight competing methods on the INbreast dataset.

As demonstrated in Table 4, we first conduct a comparison on the well-known public INbreast dataset against eight competing methodologies. These methods include ConvNext-small, proposed by Liu et al. [45] in 2022; RegNet_x_1_6gf, introduced by Xu et al. [46] in 2021; MobileNet-v2, presented by Sandler et al. [47] in 2019; EfficientNet-b0 and EfficientNet-b3, proposed by Tan et al. [48] in 2019; ResNext101_32×8d, introduced by Xie et al. [49] in 2017; and ResNet50 and ResNet101, proposed by He et al. [50] in 2016. Additionally, we plot Figure 8, which illustrates the ROC (Receiver Operating Characteristic) curves of our FV-Net model compared with these eight competing approaches, visually representing the classification performance of each method. To ensure fairness, the comparison of all models is conducted under uniform conditions involving data augmentation, data preprocessing techniques, training strategies, and Data Division methods. Notably, our proposed FV-Net model excels over all compared methods in terms of both accuracy and AUC, achieving an accuracy of 87.34% and an AUC of 0.9322. This further substantiates the effectiveness of our proposed Cross-Mammogram Dual-Pathway Attention Module and Bilateral-Mammogram Contrastive Joint Learning in proficiently mining the latent correlative and distinctive features across bilateral mammogram views.

**Figure 9:**
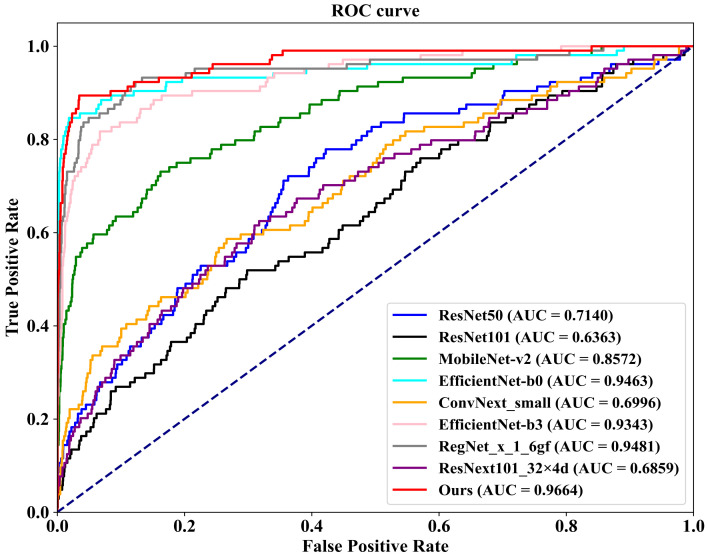
The comparison of ROC curves for the classification performance of FV-Net and eight competing methods on a 20% independent test set.

Additionally, we conduct evaluations on a 20% independent test set, as shown at the bottom of Table 4. In comparison with the other eight methods, our proposed FV-Net model achieves the best performance in both accuracy and AUC, reaching 98.02% and 0.9664, respectively. To more clearly demonstrate the comparative performance of all models, Figure 9 presents the ROC curves of our method against the other competitors on the 20% independent test set.

Furthermore, as clearly demonstrated in Table 4, compared with some of the other competing methods, our proposed FV-Net model has a smaller number of parameters (71.57 M), yet it still maintains the best performance on two different test sets. This underscores the efficacy of our meticulously designed Cross-Mammogram Dual-Pathway Attention Module and Bilateral-Mammogram Contrastive Joint Learning module, which significantly optimize model performance even with a smaller parameter count, thus confirming the superior efficiency of our model in terms of parameter utilization. This advantage is particularly valuable in clinical settings with limited computational resources, as it reduces the computational burden without sacrificing diagnostic accuracy.

**Figure 10:**
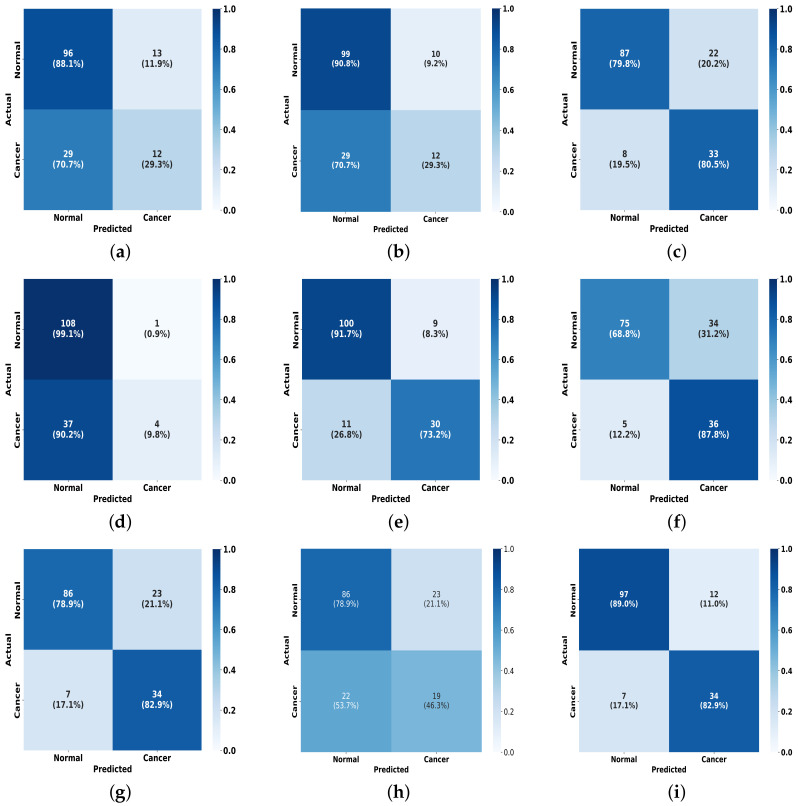
A comparison of the confusion matrices for our method against other methods on the INbreast dataset. (**a**) ResNet50; (**b**) ResNet101; (**c**) EfficientNet-b0; (**d**) ConvNext_small; (**e**) EfficientNet-b3; (**f**) Reg- Net_x_1_6gf; (**g**) MobileNet-v2; (**h**) ResNext101_32x4d; (**i**) Ours.

To further present the experimental results, as illustrated in Figure 10, we plot a visual comparison of the confusion matrix on the INbreast dataset for the FV-Net model versus the other eight methods. It is evident that our model outperforms in effective classification capability when compared to the others. This outstanding classification performance can be attributed to the effective combination of the CMDPA and BMCJL. Moreover, across both datasets, our methodology yields significant performance enhancements relative to the baseline model and consistently outperforms all other methodologies assessed. This unequivocally demonstrates that our approach not only elevates the model’s capacity for classification but also exhibits certain generalization capabilities.

**Figure 11:**
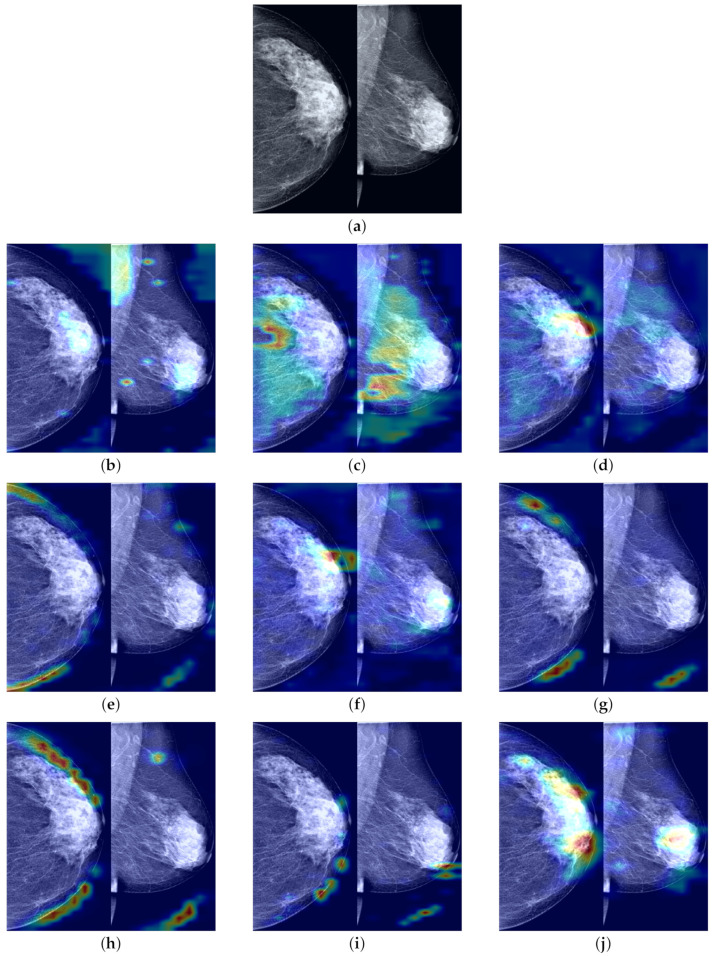
Visual comparison of the most suspicious malignant lesion areas predicted by different models. (**a**) Raw image; (**b**) ResNet50; (**c**) ResNext101_32x4d; (**d**) EfficientNet-b0; (**e**) ConvNext_small; (**f**) Efficient- Net-b3; (**g**) RegNet_x_1_6gf; (**h**) MobileNet-v2; (**i**) ResNet101; (**j**) Ours.

To demonstrate that our proposed FV-Net model has higher lesion area recognition accuracy compared to other assessed model methods, we employ Grad-CAM technology. This technique generates heatmaps based on the output feature maps of the last convolutional layers of different models and is used to conduct comparisons on the INbreast test set. The comparison results in Figure 11 clearly show the heatmaps of different models: models such as ResNet101, ConvNext_small, MobileNet-v2, and RegNet_x_1_6gf mainly focus on the edges or background areas of the breast; the EfficientNet-b0 and EfficientNet-b3 models perform slightly better, focusing on the edges of the lesion; additionally, the attention of ResNext101_32×4d widely disperses across normal breast tissue areas, while the focal areas of ResNet50 sporadically concentrate within normal breast tissue areas. In contrast, the FV-Net model identifies breast lesion areas more concentratedly and comprehensively, with its focus area almost overlapping with the entire lesion area, thereby achieving a more reasonable and precise diagnosis of breast cancer.

## 6. Conclusions

In this paper, we employ deep learning techniques to classify and diagnose normal and cancerous cases using bilateral mammograms. We preprocess the original dataset to obtain updated samples, which enhances the model’s performance in early breast cancer screening and better conforms to actual clinical diagnostics. We innovatively propose a bilateral breast four-view feature interaction model named FV-Net, containing two key components aimed at enhancing the classification capabilities of mammographic images. First, the Cross-Mammogram Dual-Pathway Attention Module (CMDPA) method achieves the association matching of the local features of each breast with the local features of the contralateral breast’s global region. This approach not only effectively mitigates the negative impact of misalignment issues in inter-breast feature matching but also captures the consistency and complementarity information between the bilateral breasts. Furthermore, the Bilateral-Mammogram Contrastive Joint Learning (BMCJL) module, by processing the recombined feature maps after bilateral breast associative learning, further enhances the correlation between similar features and increases the distinguishability of dissimilar features. This module significantly enhances the model’s ability to recognize distinct features and learn richer information on similar features. Extensive experiments conducted on test sets show that each key component of our proposed FV-Net model effectively enhances the model’s performance. Furthermore, we use Grad-CAM to conduct an interpretability analysis of the proposed method and the compared models, visualizing the regions of interest during the models’ decision-making process. This not only demonstrates the credibility and accuracy of our proposed model in identifying lesion areas but also more fully illustrates the effectiveness of the component methods we propose. The study results indicate that on a 20% independent test set, the accuracy reaches 98.02% with an AUC of 0.9664; on the INbreast test set, the accuracy reaches 87.34% with an AUC of 0.9322. Notably, our method surpasses the performance of various existing competing approaches.

## Figures and Tables

**Table 1:** Distribution of data categories without upsampling.

Dataset Name	Number of Cases with Both Breasts Normal	Number of Cases with Both Breasts Cancerous	Number of Cases with Cancer in Only One Breast	Total
Original dataset	5790	692	468	6950
Training dataset	4629	555	376	5560
Test dataset	1161	137	92	1390

**Table 2:** Distribution of data categories after upsampling.

Dataset Name	Number of Cases with Both Breasts Normal	Number of Cases with Both Breasts Cancerous	Number of Cases with Cancer in Only One Breast	Total
Training dataset	4629	2749	1880	9258
Test dataset	1161	137	92	1390

**Table 3:** Model variants’ performance on INbreast dataset.

Preprocessing Module	CMDPA	BMCJL	ACC (%)	AUC
×	×	×	68.00	0.7818
✓	×	×	80.00	0.8627
✓	✓	×	84.67	0.8877
✓	×	✓	86.67	0.8848
✓	✓	✓	87.34	0.9322

**Table 4:** Quantitative comparison of different methods on the INbreast dataset and a 20% test set.

Methods	View Approach	Data Division	#Params (M)	ACC (%)	AUC
Dataset: INbreast					
ResNet-50 [50]	Four-view	patient	105.69	72.00	0.7075
ResNet-101 [50]	Four-view	patient	178.14	74.00	0.6657
ConvNext-small [45]	Four-view	patient	190.90	72.67	0.7245
ResNext101_32x8d [49]	Four-view	patient	346.91	70.00	0.6699
EfficientNet-b0 [48]	Four-view	patient	21.55	80.00	0.8627
EfficientNet-b3 [48]	Four-view	patient	49.81	86.67	0.8823
MobileNet-v2 [47]	Four-view	patient	14.74	80.00	0.8841
RegNet_x_1_6gf [46]	Four-view	patient	34.75	74.00	0.9029
**Ours**	Four-view	patient	71.57	**87.34**	**0.9322**
Dataset: 20% test set					
ResNet-50 [50]	Four-view	patient	105.69	96.22	0.7140
ResNet-101 [50]	Four-view	patient	178.14	96.19	0.6363
ConvNext-small [45]	Four-view	patient	190.90	96.15	0.6996
EfficientNet-b0 [48]	Four-view	patient	21.55	96.94	0.9463
EfficientNet-b3 [48]	Four-view	patient	49.81	97.01	0.9343
MobileNet-v2 [47]	Four-view	patient	14.74	95.83	0.8572
RegNet_x_1_6gf [46]	Four-view	patient	34.75	95.97	0.9481
ResNext101_32x8d [49]	Four-view	patient	346.91	94.96	0.6859
**Ours**	Four-view	patient	71.57	**98.02**	**0.9664**

## Data Availability

The raw datasets utilized in this study include Vindr-mammo (https://physionet.org/content/vindr-mammo/1.0.0/ (accessed on 15 April 2023)), Mini-DDSM (https://www.kaggle.com/datasets/cheddad/miniddsm2?select=MINI-DDSM-Complete-PNG-16 (accessed on 15 April 2023)), and INbreast (https://www.kaggle.com/datasets/tommyngx/inbreast2012 (accessed on 10 November 2023)).
